# Association between Adolescents' Physical Activity and Sedentary Behaviors with Change in BMI and Risk of Type 2 Diabetes

**DOI:** 10.1371/journal.pone.0110732

**Published:** 2014-10-23

**Authors:** Paul H. Lee

**Affiliations:** School of Nursing, Hong Kong Polytechnic University, Hong Kong, China; Texas Tech University Health Science Centers, United States of America

## Abstract

This study aims at identifying the association between physical activity (PA) and sedentary behavior (SB) patterns during adolescents on the future increase in BMI and risk of diabetes during young adulthood. A total of 3,717 participants aged 11 to 21 at baseline who completed Waves I (1994–1995), II (1996), III (2001–2002), and IV (2008) surveys of the National Longitudinal Study of Adolescent Health (Add Health) were analyzed. Physical activity and sedentary behavior patterns were assessed using an interviewer-administered questionnaire at Waves I, II, and III. A participant was classified as having diabetes at Wave IV according to WHO guidelines. The *k*-means cluster analysis was used to identify the number of PA and SB patterns assessed using interviewer-administered questionnaire. The *k*-means cluster analysis identified three clusters; 575 (15.5%), 2,140 (57.6%), and 1,002 (27.0%) participants belonged to the low PA high SB (LPAHSB), the LPALSB, and the HPALSB cluster respectively. Relative to the LPALSB cluster, the HPALSB cluster had lower increase in BMI from Wave III to Wave IV (*P* = 0.03), whereas the difference between LPAHSB cluster and LPALSB cluster was not significant (*P* = 0.09). The odds of developing diabetes at Wave IV was significant for the LPAHSB cluster (OR = 1.69, 95% CI = 1.04, 2.75) but not significant for the HPALSB cluster (OR = 0.87, 95% CI = 0.52, 1.47) relative to the LPALSB cluster. To conclude, PA but not SB during adolescence predicted change in BMI during young adulthood. SB but not PA during adolescence predicted type 2 diabetes during young adulthood.

## Introduction

Diabetes is becoming more serious worldwide. A recent systematic review showed that from 1990 to 2010, the global disability-adjusted life year and global years lived with disability attributed to diabetes have increased by 69% [1] and 68% [2], respectively. High BMI and low physical activity are two well-known risk factors of type 2 diabetes, yet they are still epidemic. High BMI and low physical activity are ranked second and sixth, respectively, in the global risk factors of disability-adjusted life-years in high-income North America [3]. Type 2 diabetes is a disease not only among older adults but also among adolescents [4] and young adults [5], partly because of their high and sustained prevalence of obesity [6] and low physical activity [7].

Physical activity is a multi-dimensional, complex behavior that involves many attributes, such as duration, frequency, intensity level, and domains (such as leisure activity, occupational activity, or transport-related activity). Multiple domains have shown different effects on health. For instance, a recent study showed that occupational and household physical activity but not transportation and leisure time physical activity were associated with metabolic syndrome [8]. Furthermore, analysis of physical activity data will become more complicated if sedentary behaviors have to be taken into account. Recent studies showed that sedentary behaviors are risk factors for mortality, independent of physical activity level [9]. Given that different domains of physical activity maybe correlated, using traditional regression analysis with physical activity is not preferred, because multicollinearity problem will arise when correlated domains of physical activity/sedentary behavior variables are adjusted. The multicollinearity problem can be avoided using cluster analysis, a simple method of identifying the number of distinct patterns and their corresponding characteristics in a sample. Cluster analysis has been used to identify patterns of self-reported physical activity [10], sedentary behaviors [11], and objectively-assessed physical activity [12].

As the interlocking effects of BMI, physical activity, and type 2 diabetes have not been fully established, this study aims to explore the longitudinal association between patterns of physical activity and sedentary behaviors during adolescence and the increase in BMI and prevalence of diabetes during young adulthood, using data from the National Longitudinal Study of Adolescent Health (Add Health), a 14-year longitudinal cohort that followed the participants from adolescence in grades 7 to 12 to young adulthood.

## Materials and Methods

### Participants

The sample of Add Health comprises 80 nationally representative high schools in the United States and one randomly chosen feeder school from the 60 high schools that without a 7^th^ grade, selected using a systematic sampling method. Overall, 79% of the schools (n = 134) agreed to participate and 90,118 adolescents in grades 7 to 12 completed an in-school questionnaire in Wave I (1994–1995) and 20,475 of them completed an in-home interview. A parent or guardian of the participants was invited to complete a parent-in-home questionnaire that included demographics, socioeconomic status, information of spouse, and relationship with child. The present analysis was based on 6,504 participants with publicly available data. Among this group of participants, 4,834, 4,882, and 5,114 completed the Wave II (1996), Wave III (2001–2002), and Wave IV (2008) interviews, respectively. At Waves I and II, only in-home interviews were conducted, while at Waves III and IV biological specimen were also collected. A total of 3,808 (58.5%) participants aged 11 to 21 (3,449 of them (90.6%) aged 13 to 17) at Wave I completed all Waves, and 62 of them were removed due to missing physical activity and sedentary behavior variables. After removal of outliers in physical activity and sedentary behaviors items (n = 29), 3,717 (97.6%) participants were included in the present analysis. The incidence of diabetes and patterns of physical activity and sedentary behaviors between the 3,717 study participants and the 2,758 excluded participants were similar, but significant differences were found in sex (45.9% vs 51.6%, *P<*0.001), smoking (20.4% vs 26.1%, *P*<0.001), and drinking (22.9% vs 30.4%, *P*<0.001). Figure S1 in File S1 shows the study design of Add Health. The data and the details of the study can be found in its official Web site, http://www.cpc.unc.edu/projects/addhealth/, and the Minimal Dataset is available at File S2. This study was approved by the Institutional Review Board at the University of North Carolina at Chapel Hill. Written consent of both the adolescent and their parent were obtained.

### Measurement

Leisure time physical activity and sedentary behaviors at Waves I to III were assessed using an interviewer-administered questionnaire. The interviews at Waves I and II included 11 items examining the frequency (Not at all, 1 or 2 times, 3 or 4 times, and 5 or more times) and duration (hours per week) of physical activity and sedentary behaviors in the week preceding the interview (shown valid and reliable [13]). The interviews at Wave III included the 11 items administered at Waves I and II plus 4 additional items that are applicable to young adults [14]. Note that for some of these activities (such as the item “In the past seven days, how many times did you engage in a hobby such as working on a collection, playing cards or board games, arts and crafts, drama, playing a musical instrument or singing with a group, or shopping just for fun?”) we cannot tell whether the nature of them are physically active or sedentary, thus were removed from further analysis. As a result, only 25 out of the available 37 items were included in the current analysis (7 at Wave I, 7 at Wave II, and 11 at Wave III). Table S1 in File S1 shows these physical activity and sedentary behaviors variables. Height and weight at Waves I and II were self-reported, whereas those at Waves III and IV were measured by the interviewers following standardized protocols (details can be found in the Add Health website, http://www.cpc.unc.edu/projects/addhealth/codebooks/wave3). BMI was calculated as weight (kg) divided by the square of height (m^2^). As the variable of interest was the change in BMI over time, according to similar studies [15], raw BMI values are reported as opposed to standardized values. Information for education level of parent-in-home was collected at Wave I. Diabetes at Waves I to II and III was identified by parent-in-home and participants themselves respectively. Glucose and HbA1c level at Wave IV were obtained from dried blood spot assays. The glucose measure (available in 2,894 out of the 3,717 participants) was classified as fasting if the measurement was taken at least eight hours after last meal and non-fasting otherwise. A participant demonstrated evidence of diabetes at Wave IV if any of the following is met: [16] (1) fasting glucose ≥7.0 mmol/dL, (2) non-fasting glucose ≥11.1 mg/dL, (3) hemoglobin A1c ≥48 mmol/mol (or 6.5%), (4) self-reported history of diabetes, or (5) reported taking anti-diabetic medication.

### Statistical analysis

The *k*-means cluster analysis [17] was used to identify the number of physical activity and sedentary behavior patterns in the sample using 25 physical activity and sedentary behavior variables. All these variables were standardized to a mean and a standard deviation of 0 and 1 respectively using wave-specific means and standard deviations to equalize their importance. The *k*-means cluster analysis aims to group the participants into non-overlapping clusters by minimizing the within-group sum of squares. First, *k* (a pre-specified integer) cluster centers were randomly generated. Then, participants were assigned to the cluster with the shortest distance to these cluster centers. Finally, the cluster centers were recomputed using the new cluster assignment, and these steps would be iterated until convergence was achieved. To determine the best cluster solution, within group-sum of squares for *k* = 1 to 15 were computed and the best *k* was determined using the elbow method [18]. The first two discriminant components were used [19] to characterize the clusters. The *k*-means cluster analysis using the Hartigan and Wong's algorithm [20] was performed using R 3.0.1 (R development core team).

One-way ANOVA (and Tukey post-doc test if significant) and Pearson's χ^2^ test were used to compare the differences between clusters for continuous and categorical variables, respectively. The increase in BMI from Wave III to Wave IV and the incidence of diabetes were adjusted for age, sex, smoking and drinking habits [21] at Waves I and III, education level of parent-in-home, and parental history of diabetes, using linear regression and logistic regression, respectively. The logistic regression for incidence of diabetes excluded participants with diabetes history at Waves I to III (n = 32) and was further adjusted for BMI at Wave I. Given that the BMI were different between the clusters (see the Cluster profile: demographic characteristics, socioeconomic status, lifestyles, and biomarkers subsection below for details), for the increase in BMI from Wave III to Wave IV, the same analysis was repeated on non-overweight (BMI<25) participants. Smoking and drinking habits at Wave II were not adjusted to avoid multicollinearity problem (Kappa  = 0.61 and 0.49 for smoking and drinking respectively, both *p*<0.001). These analyses were performed using IBM-SPSS version 20.0.

### Sensitivity analysis

To confirm the association of PA, SB on increase in BMI and type 2 diabetes, regressions were fitted using the original PA and SB variables as independent variable and change in BMI and type 2 diabetes as dependent variable, with the same set of confounders adjusted. Another set of logistic regression with diabetes cases at Waves II and III (n = 7) included were performed.

## Results

### Cluster analysis

**Figure 1:**
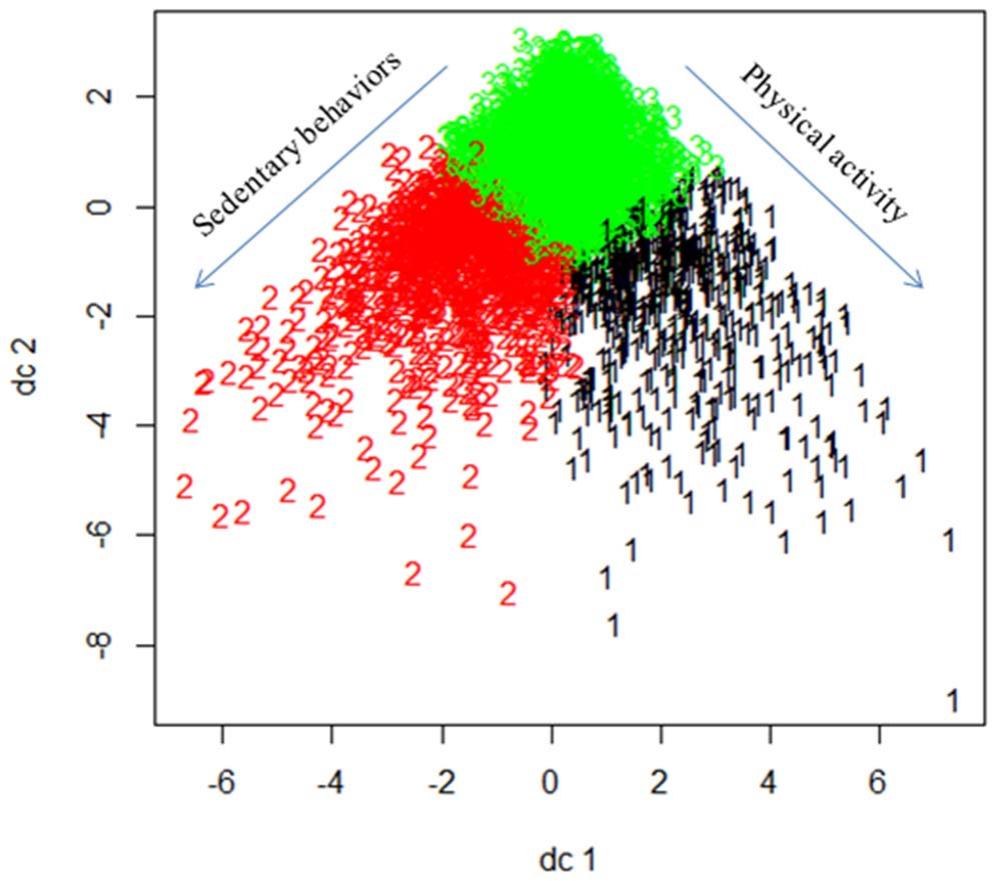
Plot of all participants by clusters on the first two discriminant components, dc1 (representing physical activity at Waves I and III and sedentary behaviors at Waves I and II) and dc2 (representing physical activity at Waves II and III and sedentary behaviors at Waves I and III).

Figure S2 in File S1 shows the within-group sum of squares of cluster solutions from *k* = 1 to 15. As a substantial reduction of within-group sum of squares from *k* = 2 to 3 were observed, a three-cluster solution was deemed appropriate. The *k*-means cluster analysis identified three clusters; 575 (15.5%), 1 002 (27.0%), and 2 140 (57.6%) participants belonged to clusters 1, 2, and 3 respectively. Table S1 in File S1 shows the loadings of the first two discriminant components of the physical activity and sedentary behavior variables. According to the loadings, the first and second components were labeled “exercise frequency” and “sitting time”. Figure 1 shows the plot of all participants on the first two discriminant components. Note that participants in cluster 1 had more sitting time, and hence, this cluster was labeled as “LPAHSB (low physical activity, high sedentary behavior) cluster”. Compared with cluster 2, participants in clusters 3 had more sitting time and at the same time exercise more frequently, and hence, clusters 2 and 3 were labeled as “LPALSB (low physical activity, low sedentary behavior) cluster” and “HPALSB (high physical activity, low sedentary behavior) cluster” respectively.

### Cluster profile: physical activity and sedentary behavior

Table 1 shows the sedentary behavior time variables in the three clusters. Time spent watching TV and videos at Waves I and II among participants in the LPAHSB cluster were nearly tripled compared with participants in the other two clusters. The trend of sedentary time was also different across clusters. Participants in the LPAHSB cluster had no change in time playing computer games from Wave I to Wave III (hours per week from 8.05 to 8.87, *P* = 0.24), whereas an increase was observed for participants in the HPALSB cluster (from 2.51 to 4.85, *P*<0.001) and LPALSB cluster (from 1.44 to 3.33, *P*<0.001). Note that participants in the HPALSB cluster had longer sedentary time than those in the LPALSB cluster. However, the differences between these two clusters were of negligible effect sizes with an average Cohen's *d* effect size of 0.10 (in which a value of >0.2 indicates clinically significant effect [22]).

**Table 1:** Participants' time spent in sedentary behaviors (hours/week) in the three clusters and the total sample.

Variable	LPAHSB cluster (n = 575)	HPALSB cluster (n = 1,002)	LPALSB cluster (n = 2,140)	Total (n = 3,717)
Wave I (1994–1995)				
Watch TV	31.25 (19.75)	13.15 (10.57)	13.30 (11.38)	16.04 (14.41)
Watch videos	10.54 (11.53)	3.23 (3.75)	2.97 (3.57)	4.21 (6.24)
Play computer games	8.05 (11.48)	2.51 (4.21)***	1.44 (2.58)	2.75 (5.86)
Wave II (1996)				
Watch TV	31.81 (19.95)	11.49 (10.17)	11.24 (10.18)	14.49 (14.28)
Watch videos	9.62 (11.19)	3.23 (3.45)	2.88 (3.43)	4.02 (5.92)
Play computer games	6.66 (9.91)	2.02 (3.53)***	1.28 (2.59)	2.31 (5.09)
Wave III (2001–2002)				
Watch TV	23.18 (19.19)	9.45 (9.38)***	11.03 (9.56)	12.48 (12.44)
Watch videos	9.14 (9.79)	4.88 (5.82)***	4.01 (4.37)	5.04 (3.17)
Play computer games	8.87 (13.35)	4.85 (7.46)***	3.33 (5.67)	4.59 (8.05)

Data are presented in mean (standard deviation).

LPAHSB: low physical activity high sedentary behavior; HPALSB: high physical activity low sedentary behavior; LPALSB: low physical activity low sedentary behavior.

All t-test *P*-values between the sedentary cluster and the more active cluster <0.001.

All t-test *P*-values between the sedentary cluster and the less active cluster <0.001.

*/**/*** difference with the less active cluster significant at 0.05/0.01/0.001 level.

Table 2 shows the physical activity and sedentary behavior frequency variables in the three clusters. For a clear presentation, only frequencies and percentages of the category “five or more times” were listed. Proportion of participants engaged in sports and exercise activities in the HPALSB cluster was much higher than that in the other two clusters. At Wave I, more than half (53.7%) of the participants in the HPALSB cluster had engaged in active sports, but this number was reduced to 26.3% and 13.0% in the LPAHSB cluster and LPALSB cluster respectively. At Wave III, participants in the HPALSB cluster were more likely to engage in all sports and exercise activities items. Note that participants in the LPAHSB cluster had more frequent engagement of physical activity than those in the LPALSB cluster. However, the differences between these two clusters were of negligible effect sizes with an average OR of 1.73 and 1.43 for Wave I and Wave II respectively (in which a value of >2 indicates clinically significant effect [22]).

**Table 2:** Physical activity and sedentary behavior: Proportions of the participants in the three clusters and the total sample who had engaged five or more times in the week preceding the interview.

Variable	LPAHSB cluster (n = 575)	HPALSB cluster (n = 1,002)	LPALSB cluster (n = 2,140)	Total (n = 3,717)
Wave I (1994–1995)				
Work around the house	235 (40.9%)	447 (44.6%)	804 (37.6%)	1,486 (40.0%)
Roller-blading/cycling	41 (7.1%)	172 (17.2%)	80 (3.7%)	293 (7.9%)
Play an active sport	151 (26.3%)	538 (53.7%)	278 (13.0%)	967 (26.0%)
Exercise, jogging, or walking	155 (27.0%)	450 (44.9%)	441 (20.6%)	1,046 (28.1%)
Wave II (1996)				
Work around the house	230 (40.0%)	416 (41.5%)	787 (36.8%)	1,433 (38.6%)
Roller-blading/cycling	25 (4.3%)	51 (5.1%)	138 (6.4%)	214 (5.8%)
Play an active sport	147 (25.6%)	535 (53.4%)	259 (12.1%)	941 (25.3%)
Exercise, jogging, or walking	153 (26.6%)	400 (39.9%)	438 (20.5%)	991 (26.7%)
Wave III (2001–2002)				
Work around the house	253 (44.0%)	491 (49.0%)	1,061 (49.6%)	1,805 (48.6%)
Bike/skateboard/bance/hike/hunt	26 (4.5%)	202 (20.2%)	65 (3.0%)	293 (7.9%)
Roller-blading/skate/ski/aerobics	8 (1.4%)	102 (10.2%)	18 (0.8%)	128 (3.4%)
Play a strenuous sports	12 (2.1%)	99 (9.9%)	2 (0.1%)	113 (3.0%)
Play individual sports	12 (2.1%)	144 (14.4%)	15 (0.7%)	171 (4.6%)
Gymnastics/weightlifting	26 (4.5%)	162 (16.2%)	31 (1.4%)	219 (5.9%)
Play golf/fish/bowling/baseball	7 (1.2%)	36 (3.6%)	3 (0.1%)	46 (1.2%)
Walk for exercise	70 (12.2%)	209 (20.9%)	206 (9.6%)	485 (13.0%)

Data are presented in frequency (percentage).

LPAHSB: low physical activity high sedentary behavior; HPALSB: high physical activity low sedentary behavior; LPALSB: low physical activity low sedentary behavior.

All χ^2^ tests across the sedentary cluster, the more active cluster and the less active cluster were significant at 0.1% level.

### Cluster profile: demographic characteristics, socioeconomic status, lifestyles, and biomarkers

Table 3 shows the profile (continuous variables) of the three clusters. The mean BMI of the participants at Wave I was 22.43 kg/m^2^. The standardized BMI (Z-score) of the participants at Waves I and II were 0.37 (SD 1.02) and 0.36 (SD 1.03) respectively (CDC 2000, http://www.cdc.gov/growthcharts/percentile_data_files.htm). The HPALSB cluster has the smallest mean age and BMI at all Waves, and the LPAHSB cluster has the largest. The fasting glucose and hemoglobin A1c levels at Wave IV were the same across all clusters, whereas the non-fasting glucose level of the participants in the LPAHSB clusters was higher than those in the LPALSB clusters (*P* = 0.007). On average, the participants had an increase of 2.68 kg/m^2^ in BMI from Wave III to Wave IV, and participants in the LPAHSB clusters had the greatest increment of 3.32 kg/m^2^.

**Table 3:** Characteristics of the three clusters and the total sample (continuous variables).

Variable	LPAHSB cluster (n = 575)	HPALSB cluster (n = 1,002)	LPALSB cluster (n = 2,140)	Total (n = 3,717)
Mean age (years) (Wave I, 1994–1995)	15.05 (1.66)***	14.73 (1.58)^###^	15.30 (1.58)^††^	15.10 (1.61)
BMI (kg/m^2^) (Wave I, 1994–1995)	23.48 (5.11)***	21.84 (3.94)^##^	22.40 (4.56)^†††^	22.41 (4.52)
BMI Z-score (Wave I, 1994–1995)	0.59 (1.08)***	0.33 (0.98)^##^	0.32 (1.01)^†††^	0.37 (1.02)
BMI (kg/m^2^) (Wave II, 1996)	24.29 (5.49)**	22.54 (4.03)^#^	22.88 (4.79)^†††^	23.01 (4.75)
BMI Z-score (Wave II, 1996)	0.61 (1.11)***	0.37 (0.95)^#^	0.29 (1.04)^†††^	0.36 (1.03)
BMI (kg/m^2^) (Wave III, 2001–2002)	27.27 (6.42)***	25.61 (1.98)	25.97 (6.33)^†††^	26.07 (6.03)
BMI (kg/m^2^) (Wave IV, 2008)	31.26 (8.50)***	28.14 (6.47)^##^	29.10 (7.79)^†††^	29.16 (7.63)
Increase in BMI (kg/m^2^) from Wave III (2001–2002) to Wave IV (2008)	3.32 (4.39)***	2.44 (3.74)	2.64 (4.24)^††^	2.68 (4.14)
Glucose level (fasting, mmol/L) (Wave IV, 2008)	6.00 (1.79) (n = 94)	5.86 (1.70) (n = 139)	5.69 (1.25) (n = 250)	5.80 (1.55) (n = 483)
Glucose level (non-fasting, mmol/L) (Wave IV, 2008)	6.22 (2.30)* (n = 334)	5.95 (1.22) (n = 635)	5.93 (1.52)^†^ (n = 1,412)	5.97 (1.59) (n = 2,381)
Hemoglobin A1c (mmol/mol) (Wave IV, 2008)	52 (93)	47 (78)	42 (46)	45 (64)
Hemoglobin A1c (%) (Wave IV, 2008)	6.9 (10.7)	6.5 (9.3)	6.0 (6.3)	6.3 (8.0)

Data are presented in mean (standard deviation).

LPAHSB: low physical activity high sedentary behavior; HPALSB: high physical activity low sedentary behavior; LPALSB: low physical activity low sedentary behavior.

*/**/***difference with the more active cluster significant at 0.05/0.01/0.001 level.

#/##/### difference with the less active cluster significant at 0.05/0.01/0.001 level.

†/††/††† difference with the sedentary active cluster significant at 0.05/0.01/0.001 level.

Table 4 shows the profile (categorical variables) of the three clusters. The LPAHSB cluster and HPALSB cluster were composed mainly of male participants (58.3% and 64.3%, respectively) whereas the LPALSB cluster was composed mainly of females participants (66.0%). Participants having a parent or caregiver with bachelor degree or above were more likely to be in the HPALSB cluster. Participants in the HPALSB cluster had the healthiest lifestyles; only 12.3% and 18.6% were current smokers and binge drinkers at Wave I, whereas these numbers increased to 22.4% and 24.1% for the LPAHSB cluster, and for the LPALSB cluster the corresponding percentages were 23.7% and 24.5% respectively. Among the participants, 4.1% of their fathers and 5.3% of their mothers had diabetes. Participants having a mother with diabetes were more likely to be in the LPAHSB cluster. At Wave IV, 212 (6.5%) participants demonstrated evidence of diabetes, and those belonging to the LPAHSB cluster had the highest prevalence (11.1%).

**Table 4:** Characteristics of the three clusters and the total sample (categorical variables).

Variable	LPAHSB cluster (n = 575)	HPALSB cluster (n = 1,002)	LPALSB cluster (n = 2,140)	Total (n = 3,717)
Male ***	335 (58.3%)	644 (64.3%)	728 (34.0%)	1,707 (45.9%)
Parent-in-home had bachelor degree or above (Wave I, 1994–1995)	114 (22.1%) (n = 516)	314 (34.7%) (n = 904)	481 (25.4%) (n = 1,897)	909 (27.4%) (n = 3,317)
Current smoker (Wave I, 1994–1995)***	109 (22.4%) (n = 486)	107 (12.3%) (n = 868)	440 (23.7%) (n = 1,857)	656 (20.4%) (n = 3,211)
Binge drinker (Wave I, 1994–1995)**	137 (24.1%) (n = 569)	185 (18.6%) (n = 994)	520 (24.5%) (n = 2,119)	842 (22.9%) (n = 3,682)
Current smoker (Wave II, 1996)***	130 (22.7%) (n = 572)	134 (13.4%) (n = 997)	454 (21.4%) (n = 2,126)	718 (19.4%) (n = 3,695)
Binge drinker (Wave II, 1996)	154 (27.2%) (n = 567)	260 (26.3%) (n = 990)	605 (28.6%) (n = 2,115)	1,019 (27.8%) (n = 3,672)
Current smoker (Wave III, 2001–2002)***	230 (50.7%) (n = 493)	349 (40.8%) (n = 855)	880 (47.5%) (n = 1,853)	1,479 (46.2%) (n = 3,201)
Binge drinker (Wave III, 2001–2002)***	273 (48.1%) (n = 567)	608 (61.6%) (n = 987)	988 (47.0%) (n = 2,102)	1,869 (51.1%) (n = 3,656)
Overweight (BMI ≥25, Wave III, 2001–2002)***	300 (55.8%) (n = 538)	445 (46.2%) (n = 964)	948 (46.5%) (n = 2,037)	1,693 (47.8%) (n = 3,539)
Overweight (Wave IV, 2008)***	362 (75.3%) (n = 481)	546 (63.0%) (n = 866)	1,209 (65.4%) (n = 1,850)	2,117 (66.2%) (n = 3,197)
Indication of diabetes by parent (Wave I, 1994–1995)	4 (0.8%) (n = 522)	0 (0.0%) (n = 913)	10 (0.5%) (n = 1,917)	14 (0.4%) (n = 3,352)
Self-reported diabetes (Wave III, 2001)	9 (1.6%)	4 (0.4%)	24 (1.1%)	37 (1.0%)
Anti-diabetic medication use (Wave IV, 2008)**	10 (2.1%) (n = 487)	2 (0.2%) (n = 877)	34 (1.8%) (n = 1,874)	46 (1.4%) (n = 3,238)
Evidence of diabetes (Wave IV, 2008)***	54 (11.1%) (n = 487)	36 (4.1%) (n = 877)	122 (6.5%) (n = 1,874)	212 (6.5%) (n = 3,238)
Diabetes (father) (Wave I, 1994–1995)	26 (5.1%) (n = 505)	37 (4.1%) (n = 899)	72 (3.9%) (n = 1,866)	135 (4.1%) (n = 3,270)
Diabetes (mother) (Wave I, 1994–1995)*	38 (8.1%) (n = 469)	39 (4.5%) (n = 868)	89 (5.0%) (n = 1,785)	166 (5.3%) (n = 3,122)

Data are presented in frequency (percentage).

LPAHSB: low physical activity high sedentary behavior; HPALSB: high physical activity low sedentary behavior; LPALSB: low physical activity low sedentary behavior.

Binge drinker defined as drinking five or more drinks in a row over the past 12 months. Evidence of diabetes at Wave IV included fasting glucose ≥7.0 mg/dL, non-fasting glucose ≥11.1 mg/dL, hemoglobin A1c ≥48 mmol/mol (or 6.5%), self-reported history of diabetes, or reported taking anti-diabetic medication.

*/**/***χ^2^ test significant at 0.05/0.01/0.001 level.

**Figure 2:**
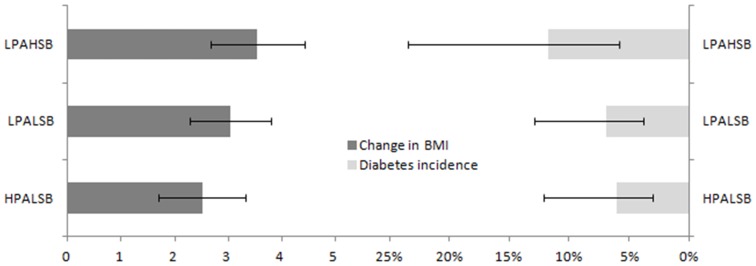
Adjusted change in BMI and incidence of diabetes of the three clusters (LPAHSB: low physical activity high sedentary behavior; HPALSB: high physical activity low sedentary behavior; LPALSB: low physical activity low sedentary behavior).

Following adjusting for age, sex, smoking and drinking habits at Waves I and III, education level of parent-in-home, and parental history of diabetes, participants in the LPAHSB cluster, the HPALSB cluster, and the LPALSB cluster had an average BMI increment of 3.55 kg/m^2^ (95% CI = 2.68,4.42), 2.51 kg/m^2^ (95% CI = 1.70,3.32), and 3.04 kg/m^2^ (95% CI = 2.28,3.79) respectively (Figure 2, left). Relative to the LPALSB cluster, the HPALSB cluster had lower increase in BMI from Wave III to Wave IV (difference  = −0.53, 95% CI = −1.01,−0.04, *P* = 0.03), whereas the difference between LPAHSB and LPALSB cluster was not significant (difference  = 0.51, 95% CI = −0.07,1.10, *P* = 0.09) (Table S2 in File S1). Similar findings were obtained if only non-overweight participants at Wave III were analyzed. Relative to the LPALSB cluster, the HPALSB cluster had lower increase in BMI from Wave III to Wave IV (difference  = −0.56, 95% CI = −1.08,−0.03, *P* = 0.04), whereas the difference between LPAHSB and LPALSB cluster was not significant (difference  = 0.46, 95% CI = −0.25,1.17, *P* = 0.21). Excluding participants with diabetes history at Waves I to III and adjusting for age, BMI, sex, smoking and drinking habits at Waves I and III, education level of parent-in-home, and parental history of diabetes, participants in the LPAHSB cluster, the HPALSB cluster, and the LPALSB cluster had a diabetes incidence of 11.7% (95% CI = 5.8%,23.5%), 6.1% (95% CI = 3.0%,12.1%), and 6.9% (95% CI = 3.7%,12.9%) respectively (Figure 2, right). Compared with the LPALSB cluster, the LPAHSB cluster had significantly greater odds for developing diabetes (OR = 1.69, 95% CI = 1.04,2.75, *P* = 0.03), whereas no significant difference was found for the HPALSB cluster (OR = 0.87, 95% CI = 0.52,1.47, *P* = 0.61) (Table S3 in File S1).

### Sensitivity analysis

The results of the linear regression on increase of BMI and the logistic regression on incidence of type 2 diabetes were shown in Tables S4 and S5 in File S1 respectively. The results of these two regression models confirmed that of the cluster analysis. There were five significant PA variables, but none of the SB variables were associated with the increase of BMI, on the other hand time spent on SB on Waves I and III were associated with the incidence of type 2 diabetes. The negative association between TV viewing at Wave II and type 2 diabetes may be a result of multicollinearity. All the PA variables that were associated with the incidence of type 2 diabetes had relatively high discriminant loadings on the SB component (Table S1 in File S1).

The ORs did not differ from the original logistic regression with participants having type 2 diabetes at Waves II and III removed (the OR for the LPAHSB cluster was 1.67 (95% CI = 1.03,2.72, *P* = 0.04) and the OR for the HPALSB cluster was 0.89 (95% CI = 0.54,1.49, *P* = 0.67), relative to the LPALSB cluster).

## Discussion

The cluster analysis identified three clusters of physical activity and sedentary behaviors across the three Waves. Participants in cluster 3 had low physical activity and high sedentary behaviors. Those in cluster 1 had similar level of physical activity but higher level of sedentary behaviors than cluster 3, while those in clusters 2 similar level of sedentary behaviors but higher level of physical activity than cluster 3. Note that a reduction of physical activity from adolescence to young adulthood was observed in all the three clusters, whereas a reduction of 25.8% in time spent watching TV (hours per week reduced from 31.3 to 23.2) was observed in the LPAHSB cluster. This was consistent with the previous finding with the same dataset that physical activity declined with age [14].

Current smokers at Waves I to III was less likely to be in the HPALSB cluster, and girls were more likely to be in the LPALSB cluster. Participants in the LPAHSB cluster had the greatest BMI whereas those in the HPALSB cluster had the lowest BMI. These findings were consistent with those of previous studies [11]. More than one-fourth (27.0%) of the participants belonged to the HPALSB cluster, comparable with a previous finding of 29% [23] (percentage of active high school students).

Although inadequate physical activity is a well-established risk factor of diabetes [24]–[26] and high BMI or obesity [27], very few studies analyzed the joint effect of physical activity and sedentary behaviors. For example, consistent with our finding that participants in the LPAHSB had a larger longitudinal increase in BMI, a meta-analysis of 4 randomized controlled studies in children and youth showed that decreasing sedentary behavior led to an average of 0.89 kg/m^2^ decrease in BMI [28]. However, the vast majority of these studies used the standard exposure-outcome approach treating TV viewing as exposure, BMI as outcome, and physical activity as confounder, ignoring the effect of other sedentary behaviors including time spent playing computer games and watching videos. Using a cluster analysis approach, we can see that sedentary behaviors were not associated with change in BMI. Our finding is consistent with a recent cross-sectional study on US adults examining the joint association between physical activity, sedentary behaviors, and BMI that found no association between BMI and total sedentary time [29], and this finding suggests a possibility that the association between sedentary behaviors and obesity is mediated by physical activity [26], which is usually lacking in sedentary people [30]. In addition, the observed association between TV viewing and BMI could also be mediated by unhealthy diet, which was supported by a study among US children that an additional hour of sitting was associated with 167 kcal of energy intake [31].

It is evident that sedentary behaviors and physical activity lead to type 2 diabetes with independent effects among adults [25], [30], but this study only confirmed the effect of sedentary behaviors [24] but not physical activity among adolescents. There are several explanations on this phenomenon. First, it was suggested that the association between physical activity and type 2 diabetes is mediated by body fat [25]; thus during the 14 years of follow-up only the control of body fat by physical activity was observed and a longer follow-up period is required to observe the effect between body fat and type 2 diabetes. Second, pervious study showed that blood glucose level was more strongly correlated with cardiorespiratory fitness than energy expenditure [32], therefore it is possible that cardiorespiratory fitness mediates the association between physical activity and type 2 diabetes. Third, the most commonly engaged sedentary behavior in our sample, watching TV, which comprised of 55% to 70% of the total sedentary time, was shown to be associated with unhealthy diet [33] and this can partially explain the association between sedentary behaviors and type 2 diabetes. Fourth, the follow-up period may be too short for observing the effect of physical activity on type 2 diabetes. A recent study showed that change in BMI was associated with risk of metabolic syndrome [34]. It is therefore possible that lack of physical activity leads to change in BMI, which was shown by the current study, and this group of participants, although did not have elevated risk of diabetes at Wave IV, was having higher risk of metabolic syndrome and elevated risk of type 2 diabetes at future. The findings of this study extend the literature by suggesting possible pathways of the preventive effect of physical activity on type 2 diabetes.

The strength of this study lies in the large, nationally representative cohort data with 14 years of follow-up period. Although the study participants had a slight difference in terms of demographic characteristics with the excluded participants, we believe that the results can be generalized to adolescents in US, and the biasedness induced for estimating the true effect of PA/SB on increase of BMI and incidence of type 2 diabetes will be minor. The use of cluster analysis allows identification of physical activity and sedentary behavior patterns specific in adolescents. Nevertheless, this study was not without limitations. First, physical activity and sedentary behaviors were self-reported, which was somewhat inaccurate [35], [36]. Similarly, height and weight at Waves I and II were self-reported. In addition, non-exercise physical activity such as job-induced activity was not assessed. To the best of the author's knowledge, there are only cross-sectional studies examining objectively measured physical activity and self-reported diabetes [12], and they could not infer causality. Thus, further research based on objectively measured physical activity is warranted. Also, as there were very few participants that engaged in both high volumes of sedentary behavior and physical activity and/or exercise, the joint effect of them could not be evaluated. Second, diabetes at Waves I to II and III was identified by parents and participants themselves respectively, and no blood samples were collected. Therefore, only participants with diagnosed diabetes reported could have been excluded from the regression analysis and those with undiagnosed diabetes were included which might induce bias. Also, the type of diabetes was unknown. However, the prevalence of undiagnosed diabetes in adolescents is low [5], so this limitation belongs to a minor one. Lastly, there are only limited data on dietary intake pattern data, which is a possible confounder of the association between TV watching and BMI increase [25], [30].

To conclude, relative to the participants in the LPALSB cluster, those in the HPALSB cluster at adolescence had lower increase in BMI during young adulthood but no reduced risk of type 2 diabetes, while those in the LPAHSB cluster at adolescence had higher risk of type 2 diabetes but no difference in the increase of BMI during young adulthood. For people with low levels of physical activity engagement, reducing sedentary behaviors is protective for type 2 diabetes. Future research of the effect and mechanism of reducing sedentary behaviors on the risk of type 2 diabetes is warranted.

## Supporting Information

File S1 **Supplemental Tables and Figures.**
(DOC)

File S2 **Minimal Dataset.**
(SAV)
