# Supplementary material for: Association between Adolescents' Physical Activity and Sedentary Behaviors with Change in BMI and Risk of Type 2 Diabetes
Source: PLoS One. 2014 Oct 23;9(10):e110732. doi: 10.1371/journal.pone.0110732 (PMC4207744; doi:10.1371/journal.pone.0110732)
Supplement: File S1 — Supplemental Tables and Figures. (DOC) [file pone.0110732.s001.doc]

Figure S1. Study design of the National Longitudinal Study of Adolescent Health (Add Health) survey, 1994-2008

**Sample**

80 high schools and their randomly chosen feeder schools

**Agree to participant**

134 schools, 90 118 participants

**In-home interview**

20 475 participants

**Public data**

6 504 participants

Wave I

1994-1995

**Public data**

4 834 participants

**Public data**

4 882 participants

**Public data**

5 114 participants

**In-home interview**

14 738 participants

**In-home interview**

15 197 participants

**In-home interview**

15 701 participants

Wave II

1996

Wave III

2001-2002

Wave IV

2008

Figure S2. Within-group sum of squares of the *k*-means cluster solutions, *k* from 1 to 15


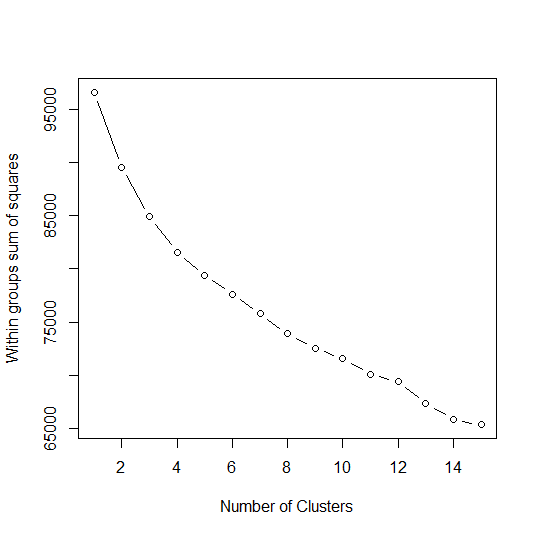


Table S1. The loadings of the first two discriminant components of the physical activity and sedentary behavior variables

| Variable | First component | Second component |
| --- | --- | --- |
| Wave I (1994-1995) |  |  |
| Work around the house | -0.002 | -0.05 |
| Roller-blading/cycling | **-0.20** | -0.20 |
| Play an active sport | **-0.21** | **-0.22** |
| Exercise, jogging, or walking | -0.12 | -0.17 |
| Hours/week watch TV | **0.25** | -0.17 |
| Hours/week watch videos | -**0.31** | **-0.32** |
| Hours/week play computer games | **0.29** | **-0.21** |
| Wave II (1996) |  |  |
| Work around the house | -0.01 | -0.02 |
| Roller-blading/cycling | **-0.23** | -0.12 |
| Play an active sport | -0.18 | **-0.31** |
| Exercise, jogging, or walking | -0.09 | -0.15 |
| Hours/week watch TV | **0.32** | **-0.39** |
| Hours/week watch videos | **0.21** | **-0.20** |
| Hours/week play computer games | **0.22** | -0.14 |
| Wave III (2001-2002) |  |  |
| Work around the house | -0.03 | 0.03 |
| Hours/week watch videos | 0.15 | -0.17 |
| Hours/week play computer games | 0.11 | **-0.20** |
| Hours/week watch TV | **0.30** | **-0.23** |
| Bike/skateboard/bance/hike/hunt | **-0.24** | -0.08 |
| Roller-blading/ skate/ski/aerobics | **-0.22** | -0.12 |
| Play a strenuous sports | **-0.28** | -0.19 |
| Play individual sports | **-0.25** | **-0.26** |
| Gymnastics/weightlifting | **-0.25** | **-0.26** |
| Play golf/fish/bowling/baseball | -0.09 | -0.005 |
| Walk for exercise | -0.06 | -0.05 |

Loadings with absolute values ≥0.2 are in bold.

Table S2. Linear regression of the association of cluster groups on increase of BMI

| Variable | Beta (95% CI) | p-value |
| --- | --- | --- |
| Physical activity and sedentary behaviors, Waves I-III (1994-2001) |  |  |
| **Cluster** |  |  |
| LPALSB | Ref |  |
| **HPALSB** | -0.53 (-1.01, -0.04) | 0.03 |
| LPAHSB | 0.51 (-0.07, 1.10) | 0.09 |
| Covariates, Wave I (1994) |  |  |
| **Sex** |  |  |
| Male | Ref |  |
| Female | -0.59 (-1.01, -0.17) | 0.01 |
| Smoker |  |  |
| No | Ref |  |
| Yes | -0.07 (-0.64, 0.50) | 0.82 |
| Binge Drinker |  |  |
| No | Ref |  |
| Yes | -0.30 (-0.82, 0.22) | 0.25 |
| **Education level of parent-in-home** |  |  |
| **8^th^ grade or less** | 2.04 (0.80, 3.28) | 0.001 |
| >8^th^ grade/didn’t graduate high school | 0.07 (-0.90, 1.04) | 0.89 |
| Business/trade/voc school instead high school | 2.23 (-0.38, 4.84) | 0.09 |
| High school graduate | 0.25 (-0.46, 0.96) | 0.49 |
| Completed a GED | 0.59 (-0.64, 1.82) | 0.35 |
| Business/trade/voc school after high school | 0.65 (-0.20, 1.49) | 0.13 |
| **College/didn’t graduate** | 1.28 (0.53, 2.03) | 0.001 |
| Graduated from college/university | 0.48 (-0.25, 1.22) | 0.20 |
| Prof training beyond 4-year college/univ | Ref |  |
| Mother has history of diabetes |  |  |
| No | Ref |  |
| Yes | -0.45 (-1.50, 0.59) | 0.39 |
| Father has history of diabetes |  |  |
| No | Ref |  |
| Yes | 0.58 (-0.33, 1.49) | 0.21 |
| **Age (years)** | -0.14 (-0.27, -0.01) | 0.04 |
| Covariates, Wave III (2001-2002) |  |  |
| Smoker |  |  |
| No | Ref |  |
| Yes | -0.18 (-0.64, 0.27) | 0.43 |
| Binge Drinker |  |  |
| No | Ref |  |
| Yes | 0.06 (-0.36, 0.49) | 0.77 |

LPAHSB: low physical activity high sedentary behavior; HPALSB: high physical activity low sedentary behavior; LPALSB: low physical activity low sedentary behavior.

Table S3. Logistic regression of the association of cluster groups on incidence of type 2 diabetes

| Variable | OR (95% CI) | p-value |
| --- | --- | --- |
| Physical activity and sedentary behaviors, Waves I-III (1994-2001) |  |  |
| Cluster |  |  |
| LPALSB | Ref |  |
| HPALSB | 0.87 (0.52, 1.47) | 0.62 |
| **LPAHSB** | 1.69 (1.04, 2.75) | 0.04 |
| Covariates, Wave I (1994) |  |  |
| Sex |  |  |
| Male | Ref |  |
| Female | 1.04 (0.69, 1.57) | 0.87 |
| Smoker |  |  |
| No | Ref |  |
| Yes | 0.82 (0.47, 1.43) | 0.48 |
| Binge Drinker |  |  |
| No | Ref |  |
| Yes | 1.05 (0.64, 1.72) | 0.85 |
| Education level of parent-in-home |  |  |
| 8^th^ grade or less | 2.58 (0.95, 7.03) | 0.06 |
| >8^th^ grade/didn’t graduate high school | 1.77 (0.73, 4.31) | 0.21 |
| Business/trade/voc school instead high school | 1.28 (0.11, 14.54) | 0.84 |
| High school graduate | 1.63 (0.76, 3.49) | 0.21 |
| Completed a GED | 0.59 (0.12, 2.90) | 0.52 |
| Business/trade/voc school after high school | 0.77 (0.29, 2.08) | 0.61 |
| College/didn’t graduate | 1.26 (0.55, 2.88) | 0.58 |
| Graduated from college/university | 1.17 (0.51, 2.70) | 0.71 |
| Prof training beyond 4-year college/univ | Ref |  |
| Mother has history of diabetes |  |  |
| No | Ref |  |
| Yes | 1.39 (0.62, 3.08) | 0.43 |
| Father has history of diabetes |  |  |
| No | Ref |  |
| Yes | 1.58 (0.80, 3.13) | 0.19 |
| Age (years) | 1.01 (0.89, 1.15) | 0.88 |
| **BMI (kg/m^2^)** | 1.08 (1.05, 1.12) | <0.001 |
| Covariates, Wave III (2001-2002) |  |  |
| Smoker |  |  |
| No | Ref |  |
| Yes | 1.16 (0.75, 1.80) | 0.51 |
| **Binge Drinker** |  |  |
| No | Ref |  |
| Yes | 0.64 (0.42, 0.97) | 0.03 |

LPAHSB: low physical activity high sedentary behavior; HPALSB: high physical activity low sedentary behavior; LPALSB: low physical activity low sedentary behavior.

Table S4. Linear regression of the association of physical activity and sedentary behaviors on increase of BMI

| Variable | Beta (95% CI) | p-value |
| --- | --- | --- |
| Physical activity and sedentary behaviors, Wave I (1994) |  |  |
| Work around the house |  |  |
| Not at all | Ref |  |
| 1 or 2 times per week | -0.19 (-1.29, 0.90) | 0.73 |
| 3 or 4 times per week | -0.21 (-1.30, 0.89) | 0.71 |
| 5 or more times per week | -0.40 (-1.51, 0.71) | 0.48 |
| Roller-blading/cycling |  |  |
| Not at all | Ref |  |
| 1 or 2 times per week | -0.09 (-0.57, 0.39) | 0.71 |
| 3 or 4 times per week | -0.47 (-1.13, 0.19) | 0.17 |
| 5 or more times per week | -0.71 (-1.46, 0.04) | 0.06 |
| Play an active sport |  |  |
| Not at all | Ref |  |
| 1 or 2 times per week | 0.05 (-0.47, 0.57) | 0.85 |
| 3 or 4 times per week | -0.03 (-0.62, 0.55) | 0.92 |
| 5 or more times per week | 0.21 (-0.39, 0.81) | 0.49 |
| Exercise, jogging, or walking |  |  |
| Not at all | Ref |  |
| 1 or 2 times per week | 0.02 (-0.58, 0.61) | 0.96 |
| 3 or 4 times per week | 0.44 (-0.19, 1.07) | 0.17 |
| 5 or more times per week | -0.19 (-0.82, 0.44) | 0.55 |
| Hours/week watch TV | 0.01 (-0.002, 0.03) | 0.09 |
| Hours/week watch videos | 0.01 (-0.02, 0.05) | 0.45 |
| Hours/week play computer games | -0.01 (-0.05, 0.02) | 0.54 |
| Physical activity and sedentary behaviors, Wave II (1995-1996) |  |  |
| Work around the house |  |  |
| Not at all | Ref |  |
| 1 or 2 times per week | 0.66 (-0.60, 1.92) | 0.31 |
| 3 or 4 times per week | 0.56 (-0.69, 1.82) | 0.38 |
| 5 or more times per week | 1.07 (-0.19, 2.34) | 0.10 |
| Roller-blading/cycling |  |  |
| Not at all | Ref |  |
| 1 or 2 times per week | -0.36 (-0.84, 0.12) | 0.15 |
| 3 or 4 times per week | -0.18 (-0.87, 0.51) | 0.60 |
| 5 or more times per week | 0.35 (-0.48, 1.17) | 0.41 |
| Play an active sport |  |  |
| Not at all | Ref |  |
| 1 or 2 times per week | -0.38 (-0.89, 0.13) | 0.14 |
| 3 or 4 times per week | -0.22 (-0.82, 0.39) | 0.49 |
| 5 or more times per week | -0.56 (-1.17, 0.05) | 0.07 |
| Exercise, jogging, or walking |  |  |
| Not at all | Ref |  |
| 1 or 2 times per week | -0.51 (-1.11, 0.10) | 0.10 |
| 3 or 4 times per week | -0.09 (-0.73, 0.56) | 0.80 |
| 5 or more times per week | -0.54 (-1.21, 0.13) | 0.12 |
| Hours/week watch TV | 0.01 (-0.01, 0.02) | 0.56 |
| Hours/week watch videos | 0.02 (-0.02, 0.06) | 0.36 |
| Hours/week play computer games | 0.02 (-0.03, 0.06) | 0.43 |
| Physical activity and sedentary behaviors, Wave III (2001-2002) |  |  |
| **Work around the house** |  |  |
| Not at all | Ref |  |
| **1 per week** | -1.47 (-2.63, -0.31) | 0.01 |
| **2 per week** | -1.03 (-2.00, -0.06) | 0.04 |
| 3 per week | -0.55 (-1.48, 0.37) | 0.25 |
| **4 per week** | -1.09 (-2.05, -0.14) | 0.03 |
| 5 per week | -0.38 (-1.39, 0.62) | 0.46 |
| 6 per week | -0.50 (-1.67, 0.68) | 0.41 |
| 7 or more per week | -0.65 (-1.54, 0.25) | 0.16 |
| Hours/week watch videos | 0.02 (-0.01, 0.05) | 0.11 |
| Hours/week play computer games | 0.01 (-0.02, 0.03) | 0.49 |
| Hours/week watch TV | -0.002 (-0.02, 0.01) | 0.77 |
| **Bike/skateboard/bance/hike/hunt** |  |  |
| Not at all | Ref |  |
| 1 per week | 0.30 (-0.28, 0.87) | 0.31 |
| **2 per week** | 0.69 (0.10, 1.28) | 0.02 |
| 3 per week | 0.48 (-0.22, 1.18) | 0.18 |
| 4 per week | -0.18 (-1.15, 0.79) | 0.72 |
| 5 per week | -0.12 (-1.26, 1.02) | 0.84 |
| 6 per week | -0.22 (-1.87, 1.42) | 0.79 |
| 7 or more per week | 0.34 (-0.74, 1.42) | 0.54 |
| Roller-blading/ skate/ski/aerobics |  |  |
| Not at all | Ref |  |
| 1 per week | 0.32 (-0.45, 1.09) | 0.42 |
| 2 per week | -0.54 (-1.39, 0.32) | 0.22 |
| 3 per week | 0.20 (-0.78, 1.19) | 0.69 |
| 4 per week | -1.21 (-2.61, 0.18) | 0.09 |
| 5 per week | -0.16 (-1.71, 1.39) | 0.84 |
| 6 per week | -1.57 (-4.72, 1.59) | 0.33 |
| 7 or more per week | -0.41 (-2.45, 1.63) | 0.70 |
| Play a strenuous sports |  |  |
| Not at all | Ref |  |
| 1 per week | -0.13 (-0.92, 0.66) | 0.75 |
| 2 per week | 0.09 (-0.80, 0.97) | 0.85 |
| 3 per week | 0.21 (-1.01, 1.42) | 0.74 |
| 4 per week | -0.29 (-1.94, 1.37) | 0.73 |
| 5 per week | 0.67 (-1.10, 2.45) | 0.46 |
| 6 per week | 0.22 (-3.19, 3.62) | 0.90 |
| 7 or more per week | 0.09 (-1.79, 1.96) | 0.93 |
| **Play individual sports** |  |  |
| Not at all | Ref |  |
| 1 per week | -0.41 (-1.19, 0.39) | 0.32 |
| **2 per week** | -0.93 (-1.68, -0.17) | 0.02 |
| **3 per week** | -1.10 (-2.02, -0.18) | 0.02 |
| 4 per week | -0.45 (-1.62, 0.72) | 0.45 |
| 5 per week | 0.29 (-1.02, 1.61) | 0.66 |
| 6 per week | -1.54 (-3.78, 0.69) | 0.18 |
| 7 or more per week | -1.20 (-2.91, 0.51) | 0.17 |
| **Gymnastics/weightlifting** |  |  |
| Not at all | Ref |  |
| 1 per week | 0.42 (-0.44, 1.28) | 0.34 |
| 2 per week | 0.23 (-0.44, 1.28) | 0.55 |
| 3 per week | 0.48 (-0.25, 1.21) | 0.19 |
| **4 per week** | 1.14 (0.12, 2.17) | 0.03 |
| 5 per week | -0.42 (-1.65, 0.80) | 0.50 |
| 6 per week | 0.12 (-1.79, 2.03) | 0.90 |
| 7 or more per week | 0.78 (-0.81, 2.37) | 0.34 |
| **Play golf/fish/bowling/baseball** |  |  |
| Not at all | Ref |  |
| 1 per week | 0.37 (-0.27, 1.01) | 0.26 |
| 2 per week | 0.63 (-0.37, 1.63) | 0.22 |
| 3 per week | 0.45 (-1.04, 1.93) | 0.56 |
| 4 per week | 0.47 (-2.00, 2.94) | 0.71 |
| **5 per week** | -2.65 (-5.39, -0.02) | 0.048 |
| 6 per week | 1.85 (-3.85, 7.56) | 0.52 |
| 7 or more per week | -0.32 (-3.35, 2.71) | 0.84 |
| Walk for exercise |  |  |
| Not at all | Ref |  |
| 1 per week | -0.19 (-1.29, 0.90) | 0.59 |
| 2 per week | -0.21 (-1.30, 0.89) | 0.39 |
| 3 per week | -0.40 (-1.51, 0.71) | 0.27 |
| 4 per week | -0.19 (-1.29, 0.90) | 0.54 |
| 5 per week | -0.21 (-1.30, 0.89) | 0.49 |
| 6 per week | -0.40 (-1.51, 0.71) | 0.35 |
| 7 or more per week | -0.19 (-1.29, 0.90) | 0.82 |
| Covariates, Wave I (1994) |  |  |
| Sex |  |  |
| Male | Ref |  |
| Female | -0.39 (-0.88, 0.09) | 0.11 |
| Smoker |  |  |
| No | Ref |  |
| Yes | 0.05 (-0.49, 0.59) | 0.86 |
| Binge Drinker |  |  |
| No | Ref |  |
| Yes | -0.32 (-0.81, 0.17) | 0.21 |
| **Education level of parent-in-home** |  |  |
| 8^th^ grade or less | Ref |  |
| **>8^th^ grade/didn’t graduate high school** | -1.91 (-3.09, -0.73) | 0.001 |
| Business/trade/voc school instead high school | 0.60 (-2.01, 3.21) | 0.65 |
| **High school graduate** | -1.54 (-2.57, -0.50) | 0.004 |
| Completed a GED | -1.31 (-2.71, 0.10) | 0.07 |
| Business/trade/voc school after high school | -1.04 (-2.17, 0.09) | 0.07 |
| College/didn’t graduate | -0.81 (-1.88, 0.27) | 0.14 |
| **Graduated from college/university** | -1.37 (-2.44, -0.30) | 0.01 |
| **Prof training beyond 4-year college/univ** | -1.93 (-3.07, -0.80) | 0.01 |
| Mother has history of diabetes |  |  |
| No | Ref |  |
| Yes | -0.53 (-1.50, 0.44) | 0.28 |
| Father has history of diabetes |  |  |
| No | Ref |  |
| Yes | 0.71 (-0.14, 1.57) | 0.10 |
| **Age (years)** | -0.18 (-0.31, -0.05) | 0.01 |
| Covariates, Wave III (2001-2002) |  |  |
| Smoker |  |  |
| No | Ref |  |
| Yes | -0.23 (-0.67, 0.21) | 0.31 |
| Binge Drinker |  |  |
| No | Ref |  |
| Yes | 0.07 (-0.34, 0.48) | 0.74 |

Table S5. Logistic regression of the association of physical activity and sedentary behaviors on incidence of type 2 diabetes

| Variable | OR (95% CI) | p-value |
| --- | --- | --- |
| Physical activity and sedentary behaviors, Wave I (1994) |  |  |
| Work around the house |  |  |
| Not at all | Ref |  |
| 1 or 2 times per week | 1.66 (0.46, 5.99) | 0.44 |
| 3 or 4 times per week | 1.97 (0.55, 7.13) | 0.30 |
| 5 or more times per week | 1.73 (0.48, 6.30) | 0.40 |
| Roller-blading/cycling |  |  |
| Not at all | Ref |  |
| 1 or 2 times per week | 1.02 (0.58, 1.78) | 0.95 |
| 3 or 4 times per week | 1.31 (0.60, 2.84) | 0.50 |
| 5 or more times per week | 1.58 (0.70, 3.60) | 0.27 |
| Play an active sport |  |  |
| Not at all | Ref |  |
| 1 or 2 times per week | 1.07 (0.60, 1.90) | 0.83 |
| 3 or 4 times per week | 1.29 (0.68, 2.44) | 0.44 |
| 5 or more times per week | 0.90 (0.46, 1.78) | 0.76 |
| **Exercise, jogging, or walking** |  |  |
| Not at all | Ref |  |
| 1 or 2 times per week | 1.02 (0.50, 2.10) | 0.95 |
| 3 or 4 times per week | 1.05 (0.48, 2.31) | 0.90 |
| **5 or more times per week** | 2.42 (1.19, 4.93) | 0.02 |
| **Hours/week watch TV** | 1.03 (1.01, 1.04) | 0.001 |
| Hours/week watch videos | 0.98 (0.96, 1.02) | 0.32 |
| Hours/week play computer games | 1.01 (0.98, 1.05) | 0.37 |
| Physical activity and sedentary behaviors, Wave II (1995-1996) |  |  |
| Work around the house |  |  |
| Not at all | Ref |  |
| 1 or 2 times per week | 0.48 (0.14, 1.71) | 0.26 |
| 3 or 4 times per week | 0.77 (0.22, 2.67) | 0.69 |
| 5 or more times per week | 0.69 (0.20, 2.41) | 0.56 |
| Roller-blading/cycling |  |  |
| Not at all | Ref |  |
| 1 or 2 times per week | 0.94 (0.54, 1.65) | 0.84 |
| 3 or 4 times per week | 1.11 (0.51, 2.39) | 0.80 |
| 5 or more times per week | 0.25 (0.05, 1.15) | 0.07 |
| Play an active sport |  |  |
| Not at all | Ref |  |
| 1 or 2 times per week | 0.72 (0.40, 1.28) | 0.26 |
| 3 or 4 times per week | 1.10 (0.57, 2.11) | 0.78 |
| 5 or more times per week | 0.83 (0.41, 1.66) | 0.59 |
| Exercise, jogging, or walking |  |  |
| Not at all | Ref |  |
| 1 or 2 times per week | 0.64 (0.32, 1.27) | 0.20 |
| 3 or 4 times per week | 1.00 (0.49, 2.01) | 0.99 |
| 5 or more times per week | 0.67 (0.32, 1.39) | 0.28 |
| **Hours/week watch TV** | 0.98 (0.96, 1.00) | 0.03 |
| **Hours/week watch videos** | 1.04 (1.01, 1.08) | 0.02 |
| Hours/week play computer games | 0.95 (0.89, 1.01) | 0.12 |
| Physical activity and sedentary behaviors, Wave III (2001-2002) |  |  |
| Work around the house |  |  |
| Not at all | Ref |  |
| 1 per week | 0.82 (0.25, 2.72) | 0.75 |
| 2 per week | 0.68 (0.22, 1.86) | 0.45 |
| 3 per week | 0.66 (0.27, 1.65) | 0.38 |
| 4 per week | 0.79 (0.31, 2.04) | 0.63 |
| 5 per week | 0.38 (0.12, 1.22) | 0.10 |
| 6 per week | 0.62 (0.16, 2.32) | 0.48 |
| 7 or more per week | 0.63 (0.27, 1.49) | 0.30 |
| Hours/week watch videos | 1.01 (0.98, 1.03) | 0.71 |
| Hours/week play computer games | 1.01 (0.98, 1.03) | 0.55 |
| Hours/week watch TV | 1.01 (1.00, 1.03) | 0.09 |
| Bike/skateboard/bance/hike/hunt |  |  |
| Not at all | Ref |  |
| 1 per week | 1.34 (0.73, 2.48) | 0.35 |
| 2 per week | 1.43 (0.78, 2.62) | 0.25 |
| 3 per week | 0.70 (0.28, 1.74) | 0.44 |
| 4 per week | 1.56 (0.51, 4.79) | 0.43 |
| 5 per week | 1.66 (0.42, 6.52) | 0.47 |
| 6 per week | N/A |  |
| 7 or more per week | 0.84 (0.22, 3.27) | 0.80 |
| **Roller-blading/ skate/ski/aerobics** |  |  |
| Not at all | Ref |  |
| 1 per week | 0.72 (0.28, 1.85) | 0.49 |
| 2 per week | 1.11 (0.38, 3.22) | 0.84 |
| 3 per week | 0.19 (0.02, 1.55) | 0.12 |
| 4 per week | 0.69 (0.08, 5.64) | 0.73 |
| 5 per week | 1.08 (0.10, 12.10) | 0.95 |
| 6 per week | N/A |  |
| **7 or more per week** | 11.40 (1.81, 71.95) | 0.01 |
| **Play a strenuous sports** |  |  |
| Not at all | Ref |  |
| 1 per week | 0.46 (0.13, 1.63) | 0.23 |
| 2 per week | 1.32 (0.48, 3.57) | 0.59 |
| **3 per week** | 4.58 (1.53, 13.74) | 0.01 |
| 4 per week | 4.22 (0.97, 18.32) | 0.06 |
| 5 per week | N/A |  |
| 6 per week | 2.54 (0.15, 43.25) | 0.52 |
| 7 or more per week | 0.40 (0.01, 13.13) | 0.61 |
| **Play individual sports** |  |  |
| Not at all | Ref |  |
| 1 per week | 1.21 (0.52, 2.81) | 0.65 |
| **2 per week** | 0.16 (0.03, 0.84) | 0.03 |
| 3 per week | 0.14 (0.02, 1.19) | 0.07 |
| 4 per week | 0.76 (0.14, 4.00) | 0.74 |
| 5 per week | N/A |  |
| 6 per week | 2.30 (0.14, 37.86) | 0.56 |
| 7 or more per week | 0.73 (0.08, 6.48) | 0.78 |
| Gymnastics/weightlifting |  |  |
| Not at all | Ref |  |
| 1 per week | 1.07 (0.40, 2.84) | 0.89 |
| 2 per week | 0.53 (0.19, 1.53) | 0.24 |
| 3 per week | 0.69 (0.26, 1.82) | 0.45 |
| 4 per week | 0.68 (0.18, 2.60) | 0.57 |
| 5 per week | N/A |  |
| 6 per week | N/A |  |
| 7 or more per week | 0.69 (0.11, 4.20) | 0.69 |
| Play golf/fish/bowling/baseball |  |  |
| Not at all | Ref |  |
| 1 per week | 1.12 (0.52, 2.40) | 0.78 |
| 2 per week | 0.78 (0.21, 2.96) | 0.72 |
| 3 per week | 0.91 (0.17, 4.92) | 0.92 |
| 4 per week | 0.63 (0.03, 12.26) | 0.76 |
| 5 per week | 0.68 (0.01, 58.97) | 0.87 |
| 6 per week | N/A |  |
| 7 or more per week | N/A |  |
| Walk for exercise |  |  |
| Not at all | Ref |  |
| 1 per week | 1.38 (0.68, 2.78) | 0.37 |
| 2 per week | 1.57 (0.81, 3.04) | 0.18 |
| 3 per week | 1.15 (0.51, 2.61) | 0.74 |
| 4 per week | 1.30 (0.44, 3.83) | 0.64 |
| 5 per week | 0.85 (0.21, 3.41) | 0.82 |
| 6 per week | N/A |  |
| 7 or more per week | 1.56 (0.76, 3.23) | 0.23 |
| Covariates, Wave I (1994) |  |  |
| Sex |  |  |
| Male | Ref |  |
| Female | 0.88 (0.51, 1.53) | 0.65 |
| Smoker |  |  |
| No | Ref |  |
| Yes | 0.68 (0.37, 1.24) | 0.21 |
| Binge Drinker |  |  |
| No | Ref |  |
| Yes | 1.32 (0.76, 2.28) | 0.32 |
| Education level of parent-in-home |  |  |
| 8^th^ grade or less | Ref |  |
| >8^th^ grade/didn’t graduate high school | 0.90 (0.32, 2.54) | 0.84 |
| Business/trade/voc school instead high school | 0.37 (0.03, 4.36) | 0.43 |
| High school graduate | 0.82 (0.33, 2.04) | 0.67 |
| Completed a GED | 0.25 (0.04, 1.43) | 0.12 |
| Business/trade/voc school after high school | 0.48 (0.16, 1.50) | 0.21 |
| College/didn’t graduate | 0.64 (0.24, 1.72) | 0.38 |
| Graduated from college/university | 0.64 (0.24, 1.72) | 0.38 |
| Prof training beyond 4-year college/univ | 0.55 (0.18, 1.68) | 0.29 |
| Mother has history of diabetes |  |  |
| No | Ref |  |
| Yes | 1.45 (0.59, 3.57) | 0.42 |
| Father has history of diabetes |  |  |
| No | Ref |  |
| Yes | 1.94 (0.92, 4.09) | 0.08 |
| Age (years) | 1.05 (0.90, 1.23) | 0.52 |
| **BMI (kg/m^2^)** | 1.09 (1.05, 1.13) | <0.001 |
| Covariates, Wave III (2001-2002) |  |  |
| Smoker |  |  |
| No | Ref |  |
| Yes | 1.07 (0.66, 1.73) | 0.79 |
| **Binge Drinker** |  |  |
| No | Ref |  |
| Yes | 0.62 (0.39, 0.99) | 0.045 |
